# Phosphocholine cytidylyltransferase MoPct1 is crucial for vegetative growth, conidiation, and appressorium-mediated plant infection by *Magnaporthe oryzae*

**DOI:** 10.3389/fmicb.2023.1136168

**Published:** 2023-05-05

**Authors:** Zhe Xu, Qi Tong, Wuyun Lv, Yu Xiao, Zhengyi Wang

**Affiliations:** State Key Laboratory of Rice Biology and Breeding & Key Laboratory of Biology of Crop Pathogens and Insects of Zhejiang Province, Institute of Biotechnology, Zhejiang University, Hangzhou, China

**Keywords:** *Magnaporthe oryzae*, phosphatidylcholine, CDP-cho pathway, gene function, pathogenicity, histone methylation, methionine metabolism

## Abstract

Phosphatidylcholine (PC) plays crucial biological roles in eukaryotic cells. In *Saccharomyces cerevisiae*, apart from phosphatidylethanolamine (PE) methylation pathway, PC is also synthesized via CDP–choline pathway. Phosphocholine cytidylyltransferase Pct1 is the rate-limiting enzyme to catalyze the conversion from phosphocholine to CDP–choline in this pathway. Here, we report the identification and functional characterization of an ortholog of the budding yeast *PCT1* in *Magnaporthe oryzae*, named *MoPCT1*. Targeted gene deletion mutants of *MoPCT1* were impaired in vegetative growth, conidiation, appressorium turgor accumulation and cell wall integrity. Also, the mutants were severely compromised in appressorium-mediated penetration, infectious growth and pathogenicity. Western blot analysis revealed that cell autophagy was activated by the deletion of *MoPCT1* under nutrient-rich conditions. Moreover, we found several key genes in PE methylation pathway, such as *MoCHO2*, *MoOPI3*, and *MoPSD2*, were significantly up-regulated in the *ΔMopct1* mutants, indicating that a pronounced compensation effect exists between the two PC biosynthesis pathways in *M. oryzae*. Interestingly, in the *ΔMopct1* mutants, histone H3 was hypermethylated and expression levels of several methionine cycling-related genes were significantly up-regulated, suggesting that *MoPCT1* is involved in histone H3 methylation and methionine metabolism. Taken together, we conclude that the phosphocholine cytidylyltransferase coding gene *MoPCT1* plays important roles in vegetative growth, conidiation and appressorium-mediated plant infection by *M. oryzae*.

## 1. Introduction

Phosphatidylcholine (PC) is the most abundant cell membrane phospholipid that involved in various crucial biological processes (Dowd et al., 2001; Deng et al., 2008; Flis et al., 2015; Haider et al., 2018). The biosynthesis pathways of PC have been extensively studied in *Saccharomyces cerevisiae*. It is now clear that two main pathways are involved in PC biosynthesis: (i) CDP-choline (CDP-C) pathway: exogenous choline (C) is firstly phosphorylated by choline kinase Cki1 to form choline phosphate, which then is activated with CTP by phosphocholine cytidylyltransferase Pct1 to produce CDP-C. In the last step, CDP-C is catalyzed by choline phosphotransferase Cpt1 with diacylglycerol (DAG) to form PC (Dowd et al., 2001); (ii) Phosphatidylethanolamine (PE) methylation pathway: PE is methylated through three steps by the methyltransferases Cho2 and Opi3 using S-adenosyl-L-methionine (SAM) as methyl donor to synthesize PC (Dowd et al., 2001).

In the budding yeast, Pct1 (also known as Cct1) is the rate-limiting enzyme of CDP-C pathway and its function is highly conserved in eukaryotes (McMaster and Bell, 1994; Dowd et al., 2001). In *Drosophila*, mutations in Cct1 result in a number of defects on oogenesis and ovarian morphogenesis, including a loss of germline stem cell maintenance, mispositioning of the oocyte, a shortened operculum and branched ovariole phenotype (Gupta and Schüpbach, 2003). Recently, in the insect pathogenic fungus *Metarhizium robertsii*, it has been showed that deletion of *MrPCT* (homologous to *S. cerevisiae PCT1*) does not affect cellular content of total PC but impair fungal virulence and increase accumulation of triacylglycerol. Moreover, under nutrient-rich conditions, *MrPCT* negatively regulates cell autophagy (Chen et al., 2018). However, the functions of *PCT1* in plant pathogenic fungi remain largely unknown.

The filamentous ascomycete fungus *Magnaporthe oryzae* (synonym of *Pyricularia oryzae*) is the causative agent of rice blast, causing huge annual loss of rice production around the world (Talbot, 2003). In the last decades, the interaction between *M. oryzae* and host plants has been developed as an excellent model system to study fungal-plant interactions due to its scientific and economic importance (Talbot, 2003; Ebbole, 2007; Wilson and Talbot, 2009). However, roles of phospholipid metabolism in morphological development and virulence have not well investigated in the rice blast fungus. Recently, it has been reported by Liu et al. that ceramide, a kind of sphingolipid, is essential for appressorial development and acts upstream from the protein kinase C-mediated cell wall integrity pathway (Liu et al., 2019). Here, we reported the identification and functional characterization of a putative phosphocholine cytidylyltransferase encoding gene *MoPCT1* in *M. oryzae*. Targeted gene deletion mutants of *MoPCT1* were severely impaired in vegetative growth, conidiation, cell wall integrity, appressorium turgor accumulation, and plant infection. Western blot analysis revealed that cell autophagy was activated by the deletion of *MoPCT1* under nutrient-rich conditions. Also, we found that histone H3 of the *ΔMopct1* mutants was hypermethylated and methionine cycle was accelerated by enhancing the expression of methionine cycling-related genes. Our findings provide novel insights into understanding the role of PC biosynthesis during fungal development and plant infection in *M. oryzae*.

## 2. Results

### 2.1. *MoPCT1* is important for vegetative growth and conidiation in *Magnaporthe oryzae*

To investigate the roles of CDP-C pathway of PC biosynthesis in pathogenesis of *M. oryzae*, we firstly identified a putative phosphocholine cytidylyltransferase encoding gene in *M. oryzae* genome by BLAST search using *S. cerevisiae PCT1* as a guide, termed *MoPCT1*(MGG_01003). And then, we carried out a targeted gene deletion of *MoPCT1*, using the background strain Guy11. A null mutant *ΔMopct1* and a complemented transformant *ΔMopct1-C* were generated (Supplementary Figure S1).

**Figure 1:**
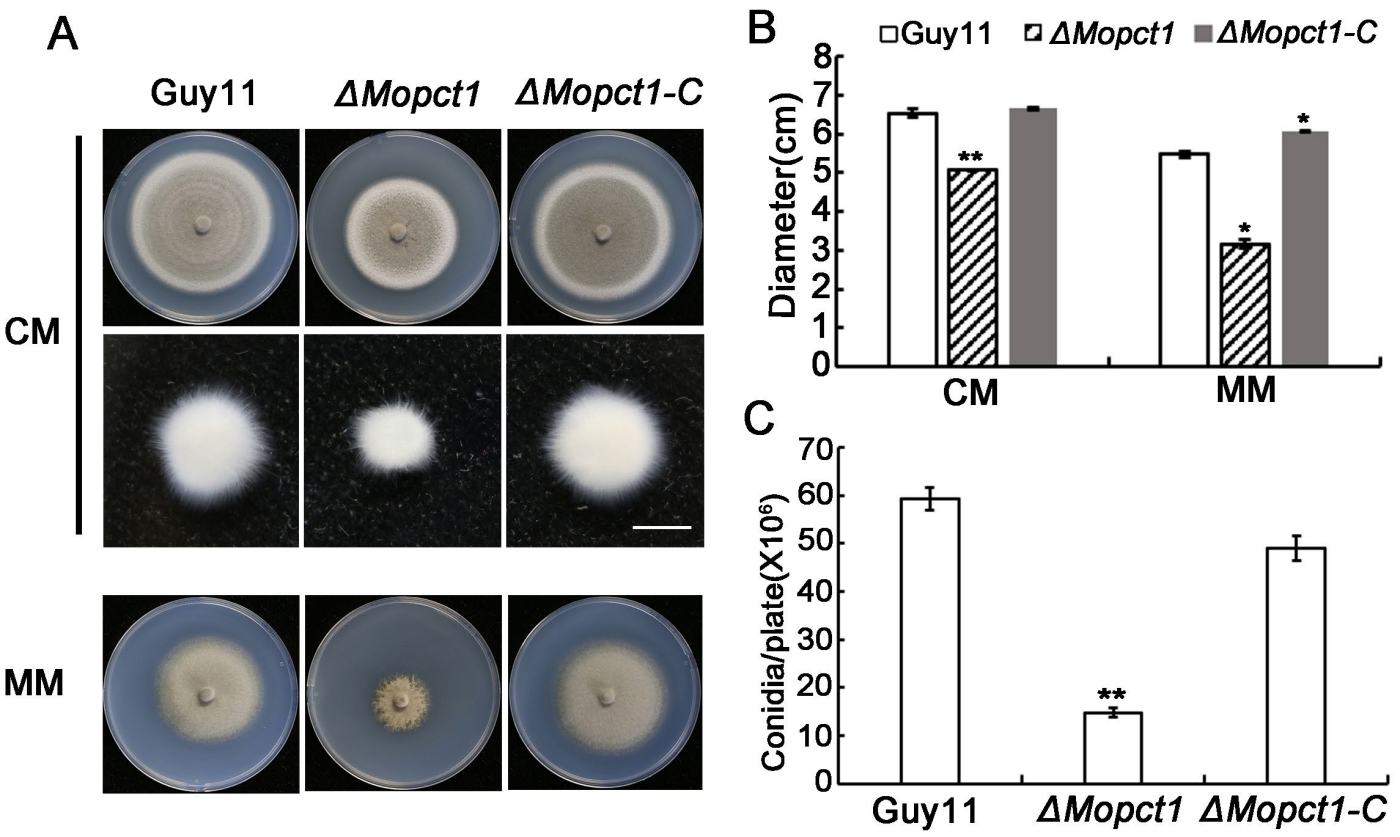
*MoPCT1* is important for vegetative growth and sporulation of *Magnaporthe oryzae*. **(A)** Colonies of the wild-type Guy11, the null mutant *ΔMopct1* and the complemented strain *ΔMopct1-C* cultured on CM (top penal) and MM (bottom penal) plates at 28°C for 10 days; mycelial plugs (5 × 5 mm) of each strain were cultured in liquid CM medium at 28°C for 48 h (middle panel). The scale bar = 5 mm. **(B)** Radial growth of each strain on CM and MM plates. **(C)** Statistical analysis of conidiation. Error bars represent standard deviation. Single asterisk indicates significant difference (*P* < 0.05). Double asterisks indicate significant difference (*P* < 0.01).

To determine the role of *MoPCT1* in growth and sporulation, the wild-type strain Guy11, the null mutant *ΔMopct1,* and the complemented transformant *ΔMopct1-C* were cultured on CM and MM medium. The *ΔMopct1* mutant showed a significant reduction in vegetative growth, compared with the wild-type Guy11 and the complemented *ΔMopct1-C* on CM or MM (Figures 1A,B). Consistently, when the hypha blocks with same size were cultured in liquid CM for 2 days, the *ΔMopct1* mutant formed much smaller mycelium pellets than those of the wild-type and the complemented strains (Figure 1A). Next, the ability to form conidia was evaluated by carefully washing the surface of 10-day-old cultures on CM plates. The *ΔMopct1* mutant produced significantly less conidia with (14.78 ± 0.96) × 10^6^ spores per plate, compared to the wild-type strain Guy11 and the complemented *ΔMopct1-C* with (59.28 ± 2.28) × 10^6^ spores per plate and (48.95 ± 2.57) × 10^6^ spores per plate, respectively (Figure 1C). These results indicate that *MoPCT1* is important for vegetative growth and conidiation of *M. oryzae*.

### 2.2. *MoPCT1* is crucial for appressorium turgor generation and plant infection by *Magnaporthe oryzae*

**Figure 2:**
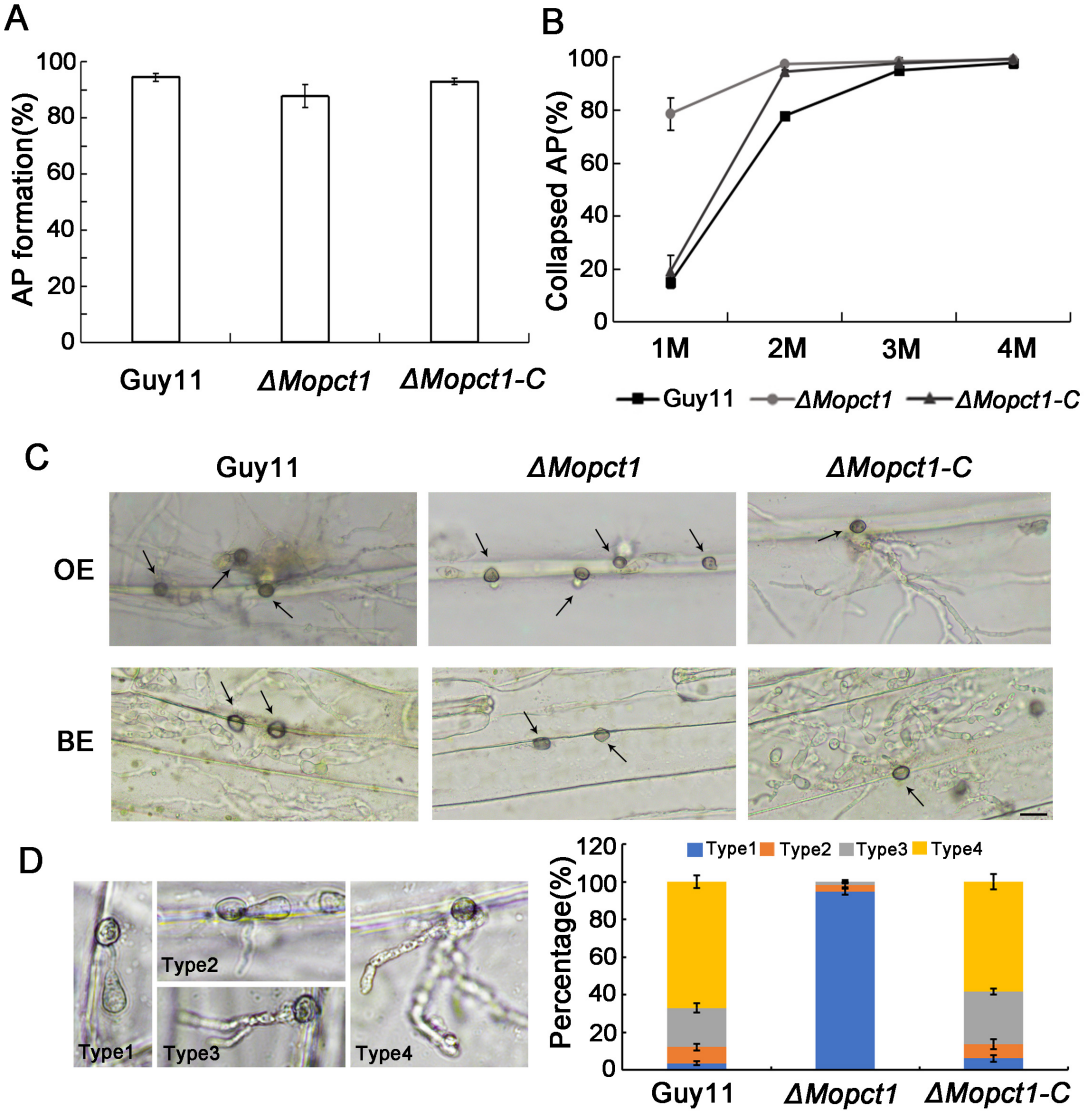
*MoPCT1* plays an important role in appressorium turgor generation and host penetration. **(A)** Appressorium formation rates. Conidia suspensions of the tested strains were incubated on hydrophobic plastic coverslips and allowed to form appressoria for 24 h. AP, appressorium. **(B)** Cytorrhysis assays. Appressoria of Guy11 and *ΔMopct1* induced for 24 h were put into gradient concentrations of glycerol (1, 2, 3, and 4 M) and the proportion of collapsed appressoria was calculated. **(C)** Penetration assays on epidermis of barley and onion. Conidial suspensions (5 × 10^4^ conidia ml^−1^) of each strain were inoculated onto the epidermis of barley or onion. After 48 h at 28°C in dark, the epidermis was observed under a light microscopy. OE, onion epidermis; BE, barley epidermis. The scale bar = 20 μm. **(D)** Statistical analysis of penetration and invasion growth on onion epidermis cells after 48 h for induction. Error bars represent standard deviations. Type 1, no penetration; type 2, invasion hyphae without branch; type 3, invasion hyphae with a single branch; type 4, invasion hyphae with branches.

In order to determine whether deletion of *MoPCT1* influences appressorium formation, we carried out appressorium induction experiments. Conidia from different strains were allowed to germinate on a hydrophobic surface. By 24 h, we observed that the appressorium formation rate of the *ΔMopct1* mutant was not significantly different from that of the wild-type Guy11 or the complemented *ΔMopct1-C* (Figure 2A). To further determine whether deletion of *MoPCT1* leads to alteration of appressorium turgor, we carried out cytorrhysis assays. Appressoria were allowed to form on plastic cover slips for 24 h, and the proportion of collapsed appressoria after exposure to different glycerol solutions was calculated. We found that, under 1 M glycerol solution, the percentages of collapsed appressoria of the wild-type and complemented strains were 14.82 ± 2.11% and 18.97 ± 6.12%, respectively, significantly lower than (78.49 ± 4.57) % of the *ΔMopct1* mutant (Figure 2B). Even when the induction time for appressorium formation was extended to 48 h, the appressorium turgor defect of the mutant was not restored (data not shown). These results indicate that *MoPCT1* is dispensable for appressorium formation but crucial for appressorium turgor generation in *M. oryzae*. Next, cuticle penetration and invasive growth assays were performed on onion and barley epidermis. By 48 hpi, most conidia of Guy11 and *ΔMopct1-C* could penetrate cuticle and form dense infectious hyphae on either onion or barley epidermis. However, most appressoria formed by *ΔMopct1* could not infect the epidermis (Figure 2C). To quantitatively evaluate fungal infectious development on onion epidermis, the infection structures were divided into four types (Figure 2D). On onion epidermis, up to 67% appressoria of Guy11 and 58% appressoria of *ΔMopct1-C* could form type 4, in contrast to 94.67% appressoria formed by the *ΔMopct1* mutant could not penetrate the epidermis cell surfaces (Figure 2D). Similar results were observed on barley epidermis (data not shown).

**Figure 3:**
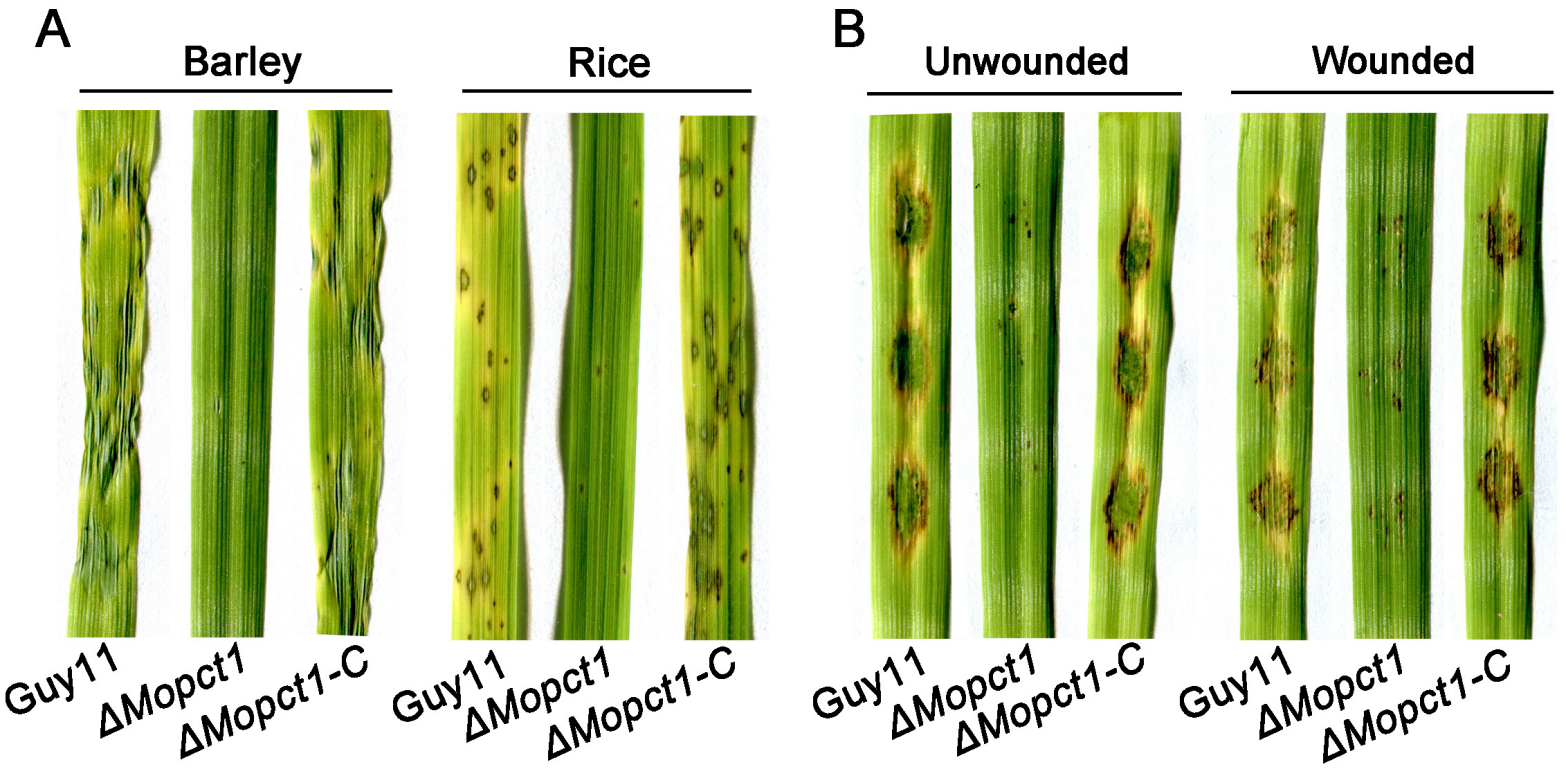
*ΔMopct1* is significantly impaired in pathogenicity. **(A)** Spray-inoculation assays. Barley and rice seedlings were spray-inoculated with 10 ml conidial suspension (5 × 10^4^ conidia ml^−1^) of each strain. Photographs were taken at 5 days post inoculation (dpi). **(B)** Barley cut leaf inoculation assays. Conidia suspensions (5 × 10^4^ conidia ml^−1^) of various strains were dropped onto barley leaf segments wounded or unwounded. Photographs were taken at 5 dpi.

To further determine whether *MoPCT1* is required for pathogenicity, conidial suspensions of the wild-type Guy11, the *ΔMopct1* mutant, and the *ΔMopct1-C* were sprayed onto 7-day-old barley or 14-day-old rice leaves, respectively. After 5 days, the wild-type strain and the complemented *ΔMopct1-C* caused numerous typical spreading lesions on the host leaves, in contrast to much less and smaller lesions caused by the *ΔMopct1* mutant (Figure 3A). In addition, the cut-leaf assays displayed that pathogenicity of the *ΔMopct1* mutant was severely compromised on intact barley leaves, even when they were abraded to remove the surface cuticle (Figure 3B). These results indicate that *MoPCT1* is required for fungal penetration and host intracellular colonization by *M. oryzae*.

### 2.3. MoPct1 localizes to the nucleus, but its nuclear localization signal is dispensable for mycelium growth, conidiation, and pathogenicity

**Figure 4:**
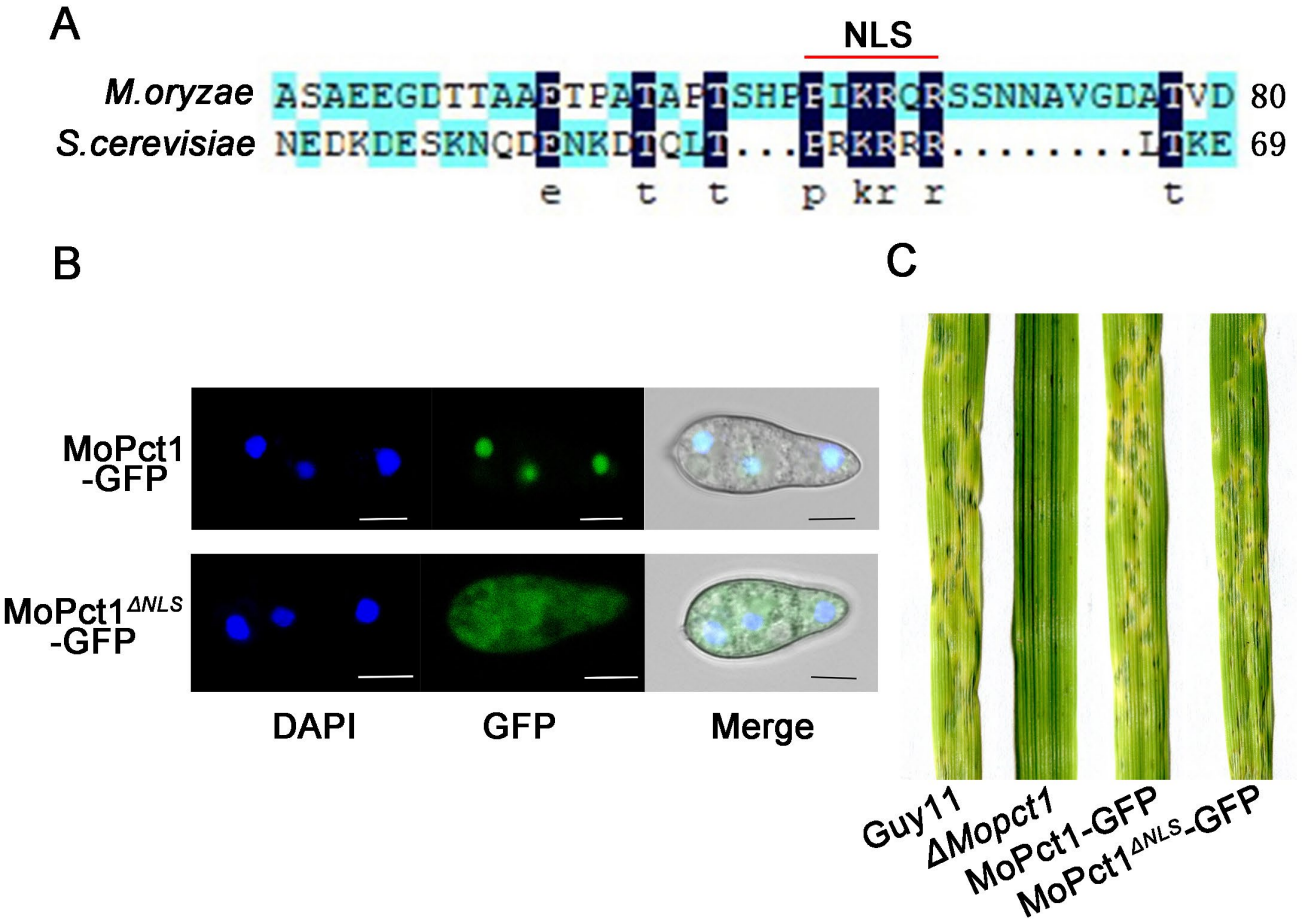
*MoPCT1* mainly localizes to the nucleus and NLS of MoPct1 is dispensable for pathogenicity by *Magnaporthe oryzae*. **(A)** The nuclear localization signal (NLS) of MoPct1 was predicted by the alignment with *Saccharomyces cerevisiae* Pct1. The red line indicates the predicted NLS of MoPct1. **(B)** GFP fluorescence was examined under a fluorescent confocal microscopy. Bars = 5 μm. **(C)** Spray-inoculation assays. Barley seedlings were spray-inoculated with 10 ml conidial suspension (5 × 10^4^ conidia ml^−1^) of each strain. Photographs were taken at 5 dpi.

In *S. cerevisiae*, Pct1 is mainly located in the nucleoplasm and nuclear membranes (MacKinnon et al., 2009). There is a conserved sequence at the N terminal of Pct1, which is presumed to be nuclear localization signal (MacKinnon et al., 2009). By protein sequence alignment analysis, we identified a hypothetical nuclear localization signal in MoPct1, named NLS (Figure 4A). To determine whether the predicted NLS of MoPct1 is required for its nuclear localization, a complete *MoPCT1* gene fragment and the fragment without the NLS were ligated with GFP and then the resulting constructs were transformed into the *ΔMopct1* mutant, respectively. Consequently, strains expressing MoPct1-GFP or MoPct1^ΔNLS^-GFP were obtained. By a laser confocal microscope, we observed that MoPct1-GFP mainly localized to nucleus of conidium cells, while MoPct1^ΔNLS^-GFP was diffusely distributed in cytoplasm (Figure 4B), indicating the NLS is required for nuclear localization of MoPct1. To investigate the effect of MoPct1 NLS on pathogenicity of the blast fungus, we carried out spray-inoculation assays on barley seedlings. Surprisingly, the strains expressing the MoPct1^ΔNLS^-GFP were fully pathogenic to barley leaves (Figure 4C). Further phenotypic analysis of the strains showed that absence of NLS signal did not affect mycelium growth and sporulation of *M. oryzae* (Supplementary Figure S2). These results suggested that the NLS of MoPct1 is essential for transportation of the protein to nucleus but dispensable for mycelium growth, conidiation and pathogenicity by *M. oryzae*.

### 2.4. *MoPCT1* is required for cell wall integrity by *Magnaporthe oryzae*

**Figure 5:**
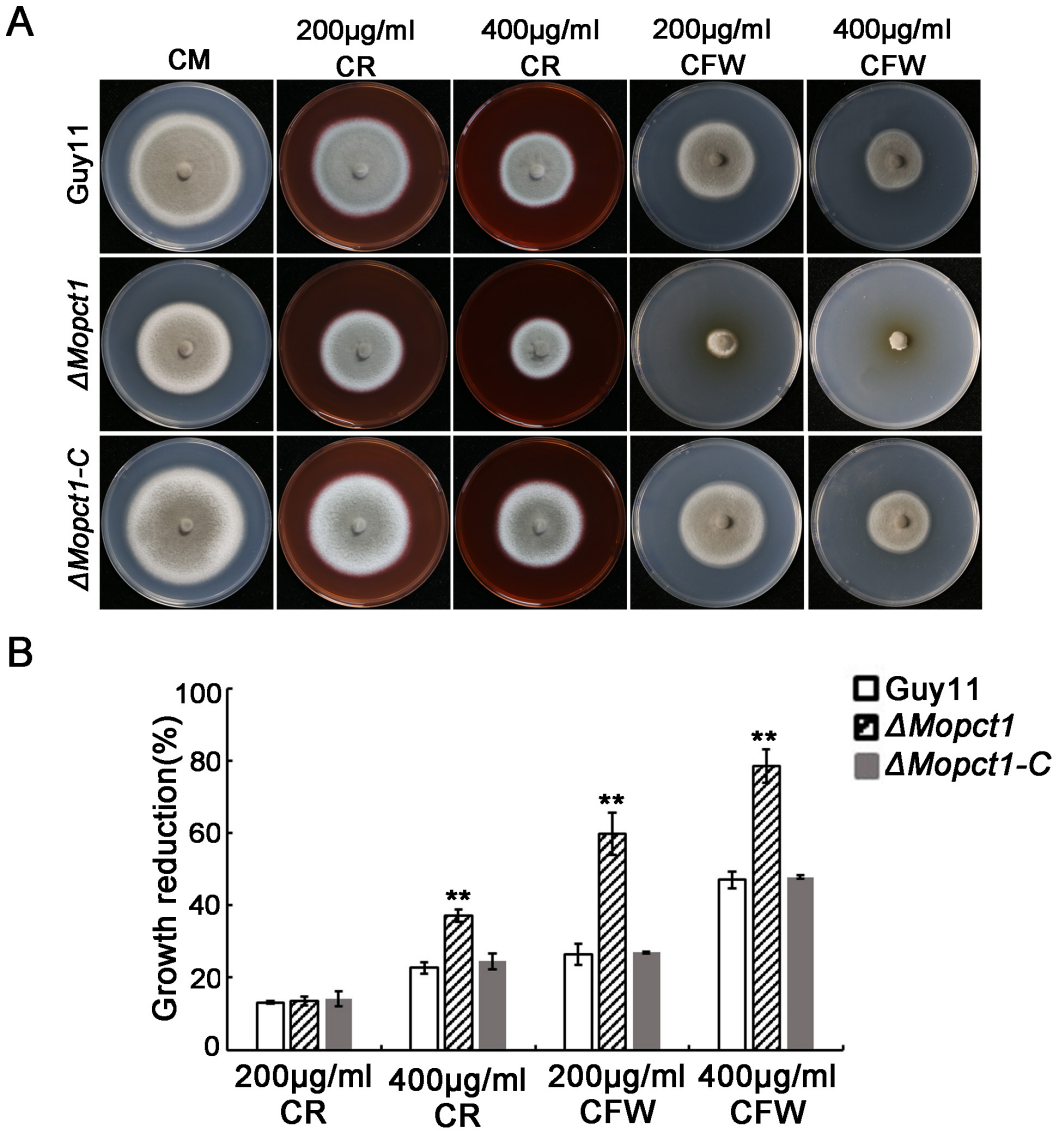
Deletion of *MoPCT1* leads to hypersensitivity to cell wall stressors. **(A)** The strains incubated on CM supplemented with Congo red (CR) or calcofluor white (CFW) at 28°C for 10 days. **(B)** Colony growth reduction rates of the tested strains on different medium. Growth reduction rate = (diameter of Guy11-diameter of a tested strain)/diameter of Guy11. Standard deviations were calculated based on three independent experiments. Error bars represent standard deviation. Double asterisks indicate significant difference (*P* < 0.01).

Cell wall integrity is essential for the maintenance of appressorium turgor (Xu et al., 1998). To determine whether knockout of *MoPCT1* affects cell wall integrity of *M. oryzae*, we measured the sensitivity of different strains to cell wall stress. The wild-type Guy11, the *ΔMopct1* mutant, and the complemented *ΔMopct1-C* were inoculated on CM medium added cell wall stressors, including Congo red (CR) and calcofluor-white (CFW). After 10 days post-inoculation, colony diameters of different cultures were measured. The results showed that the growth reduction rate of the *ΔMopct1* mutant was significantly higher than the wild-type Guy11 and the complemented *ΔMopct1-C* under the condition supplemented with 400 μg/ml CR, 200 μg/ml CFW or 400 μg/ml CFW (Figure 5), indicating that the null mutant is hypersensitive to cell wall stress and *MoPCT1* is required for cell wall integrity of *M. oryzae*.

### 2.5. Deletion of *MoPCT1* activates cell autophagy under nutrient-rich conditions

**Figure 6:**
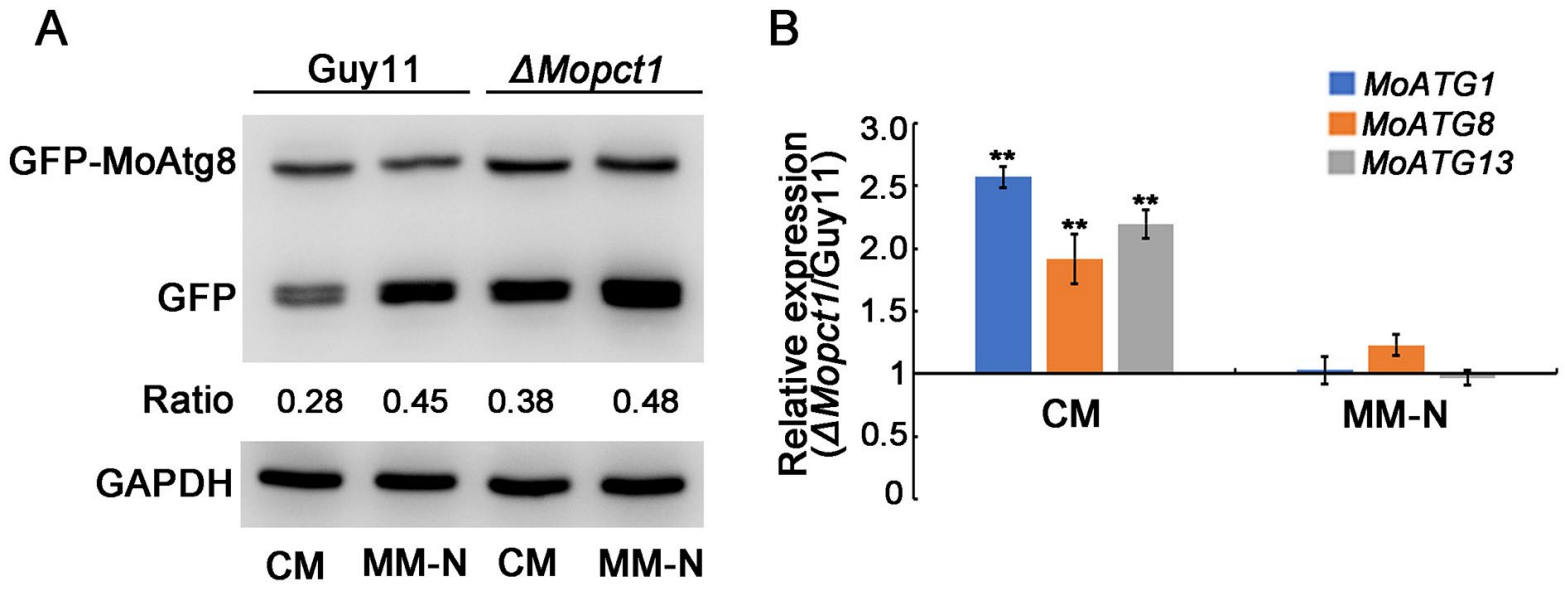
Deletion of *MoPCT1* activates cell autophagy under non-induced conditions. **(A)** GFP-MoAtg8 proteolysis assays. Immunoblot assays with total lysates from CM or MM-N cultures of various strains using anti-GFP antibody or anti-GAPDH antibody. **(B)** Expression levels of autophagy-related genes under CM or MM-N conditions in the *ΔMopct1* mutant compared to that in the wild-type Guy11. Error bars represent standard deviation. Double asterisks indicate significant difference (*P* < 0.01).

To determine whether autophagy is affected by deletion of *MoPCT1* in *M. oryzae*, we tested the degradation process of GFP-MoAtg8 by Western blot assays. The GFP-MoAtg8 fusion protein was constructed and transformed into the wild-type Guy11 and the *ΔMopct1* mutant, respectively. Mycelium of the wild-type or the mutant expressed GFP-MoAtg8 was cultured in CM liquid medium for 36 h (28°C, 180 rpm), and then transferred part of the mycelium to MM-N medium to induce cell autophagy for 8 h. All the mycelium was collected and total protein of the tested strains was extracted. Western blot analysis displayed that a clear full-length GFP-MoAtg8 band (40 kDa) and a free GFP band (26 kDa) were detected in both the wild-type Guy11 and the *ΔMopct1* mutant (Figure 6A). By calculating the ratio of free GFP to total GFP (sum of free GFP and GFP-MoATG8), we found that under CM condition, the GFP ratio of Guy11 and *ΔMopct1* was 0.28 and 0.38, respectively. The proportion of free GFP in *ΔMopct1* was significantly higher than that of Guy11, suggesting the autophagy level of the *ΔMopct1* was higher under nutrient-rich conditions than the wild-type strain Guy11. When hyphae were shifted to nitrogen starvation conditions (MM-N medium) for 8 h, an increasingly stronger free GFP band were detected both in the wild-type Guy11 and the *ΔMopct1*, indicating that deletion of *MoPCT1* does not affect the response to nitrogen starvation in *M. oryzae*.

Then we examined the expression levels of several key autophagy-related genes including *MoATG1*, *MoATG8* and *MoATG13*. The expressions of the three genes in mycelium of the *ΔMopct1* mutant were significantly up-regulated in CM condition compared with the wild-type, but no significant difference was observed in MM-N condition (Figure 6B), indicating that *MoPCT1* negatively regulates autophagy process of *M. oryzae* under nutrition-rich conditions. In addition, the expressions of the ATG-related genes during appressoria infection on barley leaves were determined by qRT-PCR. We found that only *MoATG1* expression in the mutant was significantly up-regulated (Supplementary Figure S3A).

### 2.6. Deletion of *MoPCT1* activates the PE methylation pathway of PC biosynthesis

**Figure 7:**
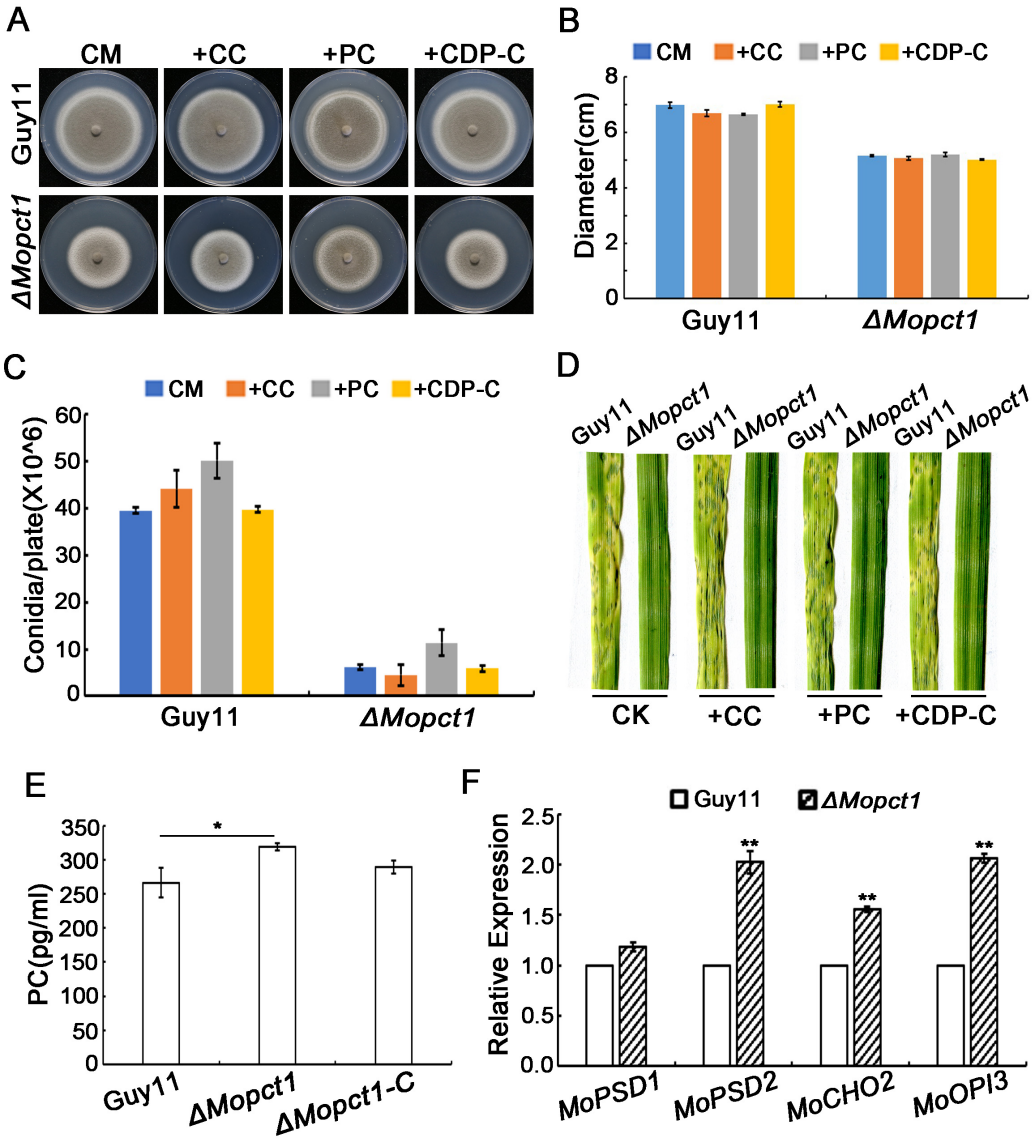
Deletion of *MoPCT1* activates the PE methylation pathway of PC biosynthesis. **(A)** Growth of the *ΔMopct1* mutant on CM added 1 mM PC, 0.1 mM CDP-C, or 1 mM CC. The plates were cultured at 28°C for 10 days. **(B)** Radial growth of each strain under different condition. **(C)** Statistical analysis of conidiation. Error bars in **(B)** and **(C)** represent standard deviation. **(D)** Spray-inoculation assays. Barley seedlings were spray-inoculated with 10 ml conidial suspension (5 × 10^4^ conidia ml^−1^) of each strain with different additions (1 mM PC, 0.1 mM CDP-C, or 1 mM CC). Photographs were taken at 5 dpi. **(E)** Bar chart of PC content for Guy11 and *ΔMopct1*. **(F)** Expression levels of the genes involved in PE methylation pathway. Means and standard deviations were calculated based on three independent experiments. Error bars represent standard deviation. Double asterisks indicate significant difference (*P* < 0.01).

To investigate the role of *MoPCT1* in PC synthesis, the Guy11 strain and the *ΔMopct1* were inoculated on CM medium supplemented with choline chloride (CC), CDP-C, or PC, respectively. Colony diameter was measured after 10 days post incubation at 28°C. The results showed that the defects in growth, asexual sporulation and pathogenicity of the *ΔMopct1* mutant were not restored by adding exogenous CC to CM medium (Figures 7A–D), indicating that the utilization of exogenous choline via CDP–C pathway for PC synthesis is blocked by the deletion of *MoPCT1*. Surprisingly, adding exogenous CDP-C or PC, which are downstream products of the reaction catalyzed by MoPct1, also failed to recover the phenotypic defects of the *ΔMopct1*(Figures 7A–D). To further test whether deletion of *MoPCT1* causes the defect in PC production, the PC content of the Guy11 strain and the *ΔMopct1* was detected by ELISA method. The results exhibited that the content of PC in *ΔMopct1 was* (318.67 ± 5.00) pg./ml, while the content of PC in Guy11 and *ΔMopct1-C* was (266.3 ± 21.39) pg./ml and (289.14 ± 9.34) pg./ml, respectively, (Figure 7E). It indicated that deletion of *MoPCT1* did not lead to reduction of PC, but caused the significant increase of PC content in *M. oryzae*. We hypothesize that the PE methylation pathway of PC synthesis may be activated in the *ΔMopct1* mutant to maintain intracellular PC synthesis. To test the hypothesis, we determined transcriptional expression of *MoCHO2* and *MoOPI3*, which encode the two key enzymes in the PE methylation pathway, as well as *MoPSD1* and *MoPSD2*, which encodes enzymes to catalyze PE synthesis by RT-qPCR. The results showed that expression levels of *MoPSD2*, *MoCHO2* and *MoOPI3* in the *ΔMopct1* mutant were significantly up-regulated (Figure 7F), indicating that the PE methylation pathway of PC synthesis is activated by the loss of *MoPCT1*.

### 2.7. Disruption of *MoPCT1* causes hypermethylation of histone H3 and acceleration of methionine metabolism cycle

**Figure 8:**
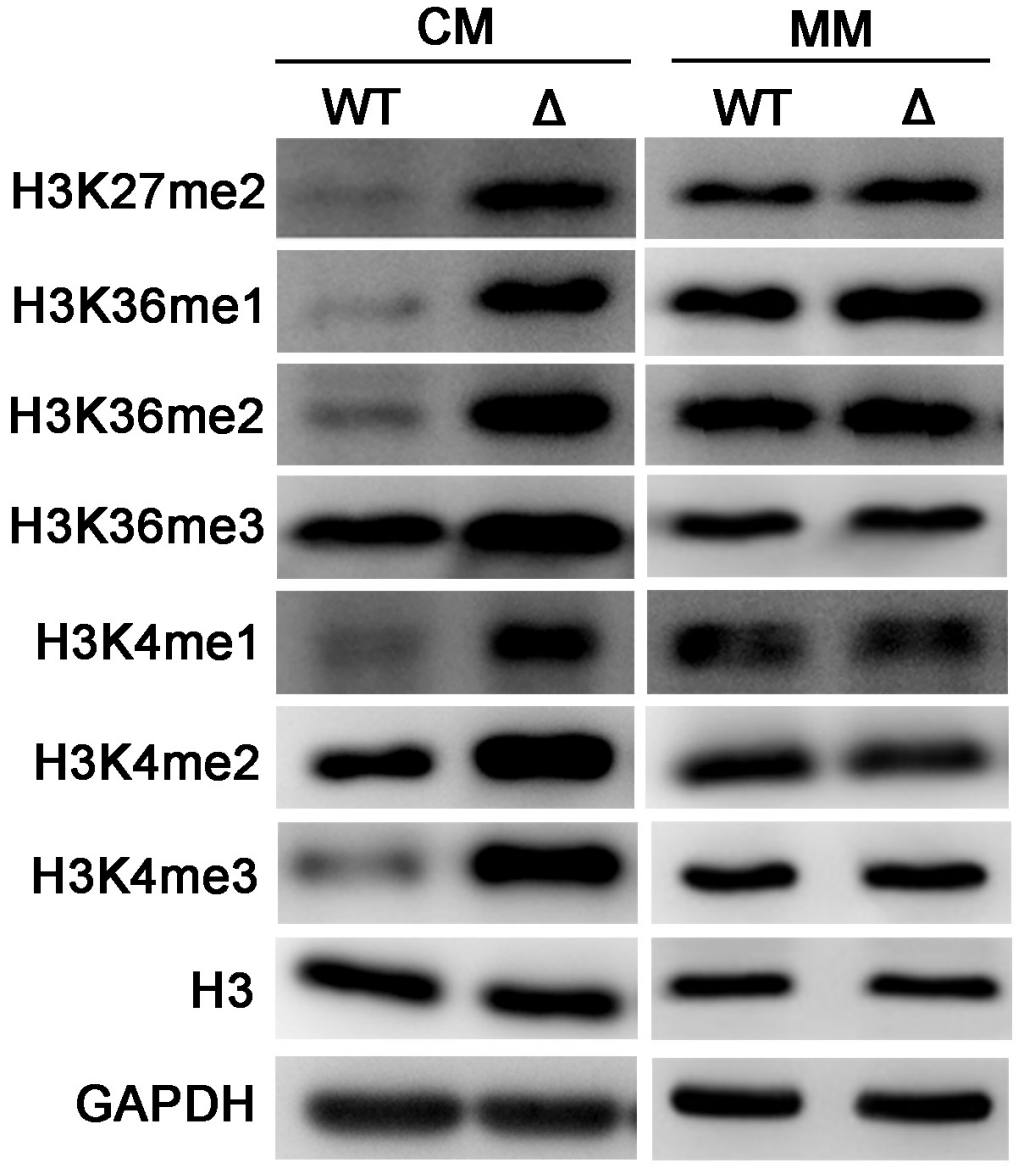
Disruption of *MoPCT1* leads to the hypermethylation of histone H3 under nutrition-rich condition. Levels of methylated H3 at specific lysine sites in Guy11 and *ΔMopct1* under CM or MM conditions. WT: Guy11; Δ: *ΔMopct1*. The data are representative of at least three independent experiments.

In *S. cerevisiae*, PE methylation is a major consumer of SAM. Deletion of *CHO2* could result in SAM accumulation, thus causes the hypermethylation of Histone H3 (Ye et al., 2017). To determine whether activation of PE methylation in the *ΔMopct1* affects H3 methylation, we measured the level of H3 methylation by Western blot assays. Unexpectedly, the methylation levels on various sites of histone H3 under CM condition notably increased in the *ΔMopct1* compared with that in the wild-type strain, such as H3K4, H3K27, and H3K36 (Figure 8). On the contrary, there were no differences of the methylation levels of histone H3 between the wild-type Guy11 and the *ΔMopct1* on MM medium (Figure 8). These results suggest that deletion of *MoPCT1* results in excess methyl flow into the histone methylation under CM condition.

**Figure 9:**
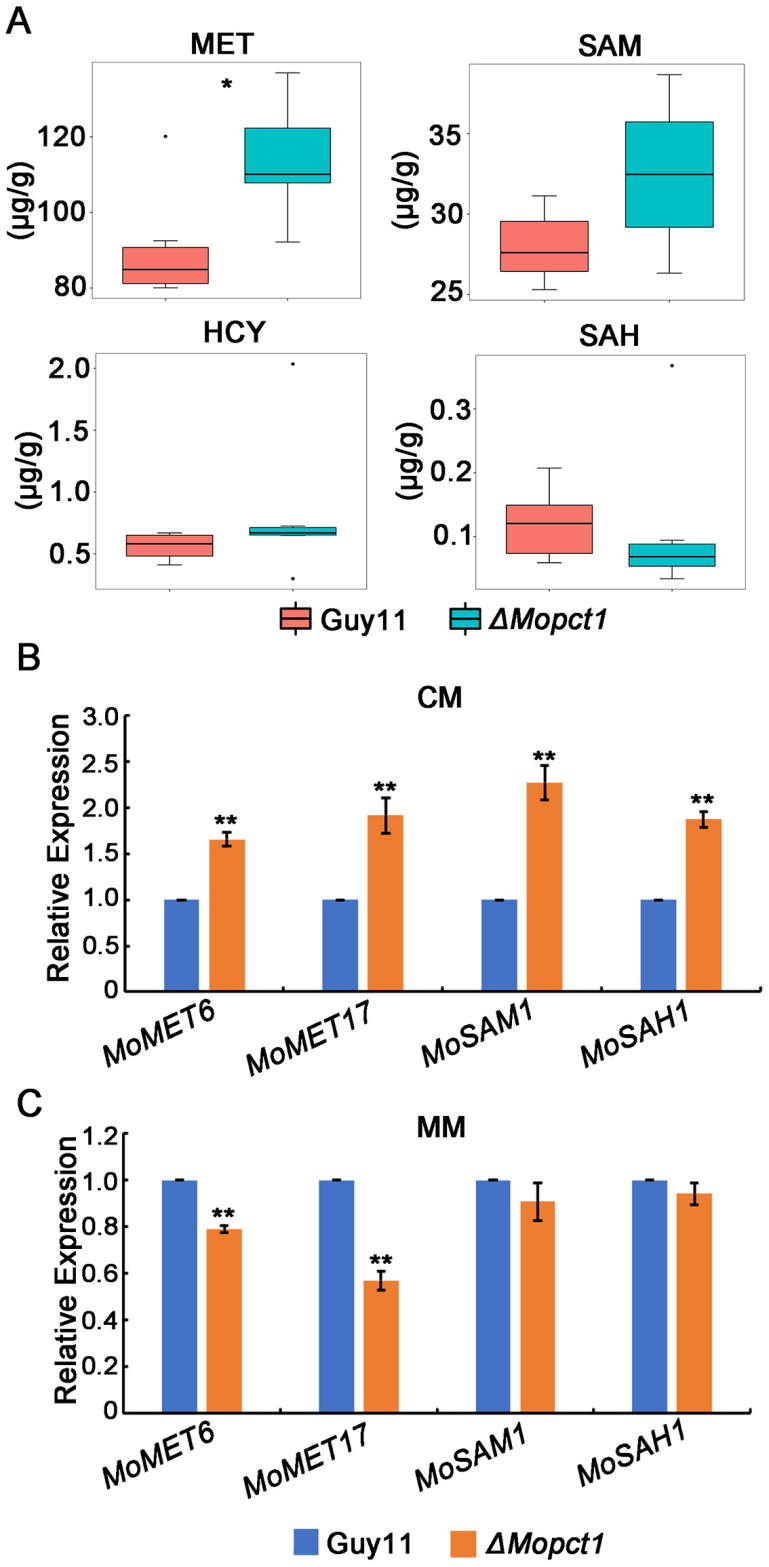
Disruption of *MoPCT1* causes acceleration of methionine metabolism cycle. **(A)** LC–MS/MS analysis. Intracellular levels of methionine-related metabolites determined by multiple reaction monitoring (MRM) using LC–MS/MS. There were 6 replicates per sample. Single asterisk indicates significant difference (*P* < 0.05). **(B)** and **(C)** RT-qPCR analysis. Expression of the genes associated with methionine metabolic cycle in different strains growth on CM **(B)** or MM **(C)** was determined. Means and standard deviations were calculated based on three independent experiments. Error bars represent standard deviation. Double asterisks indicate significant difference (*P* < 0.01).

Methyl is produced from SAM to form SAH, which is a key part of methionine metabolism cycle, and therefore SAM is the methyl donor for biological methylation modifications (Stipanuk, 2004; Brosnan and Brosnan, 2006). Since activation of the PE methylation pathway and hypermethylation of histone H3 in the *ΔMopct1* consume large amounts of methyl groups, to determine whether the methyl depletion affects SAM content and methionine cycle, we detected the contents of SAM, homocysteine (HCY), methionine (MET), and S-adenosylhomocysteine (SAH) in the *ΔMopct1* by LC/MS. The results showed that the content of HCY, SAM or SAH in the *ΔMopct1* was not statistically different from that of the Guy11 strain. However, the content of MET in the *ΔMopct1* was significantly increased (Figure 9A).

We hypothesized that the large amount of methyl provided by the *ΔMopct1* mutants may be caused by the acceleration of the entire methionine cycle. To test this hypothesis, expression levels of methionine cycling-related genes were detected by RT-qPCR. The results showed that the expression levels of *MoMET6*, *MoMET17*, *MoSAM1*, and *MoSAH1*, key genes of methionine cycling, were significantly up-regulated in mycelium of the *ΔMopct1* compared with Guy11 under CM condition (Figure 9B). Moreover, the expressions of *MoSAH1*, *MoSAH1,* and *MoMET6* during appressoria infection were up-regulated too (Supplementary Figure S3B). These results suggested that the flux of methionine metabolism is enhanced under nutrition condition. However, under the MM condition, the expressions of *MoSAM1* and *MoSAH1* in the *ΔMopct1* were not significantly different compared to Guy11, while the expressions of *MoMET6* and *MoMET17* in the *ΔMopct1* were significantly decreased (Figure 9C). These results indicated that the methionine cycle was activated in the *ΔMopct1* under nutrition-rich conditions, which may provide a large number of methyl groups for PE methylation pathway and histone H3 methylation.

## 3. Discussion

In *S. cerevisiae*, phosphocholine cytidylyltransferase Pct1 is the rate-limiting enzyme in CDP-C pathway for PC synthesis (Dowd et al., 2001). Recently, it has been reported that deletion of *MrPCT* (homologous to *PCT1*) significantly reduces the growth rate and pathogenicity in the insect pathogenic fungus *M. robertsii* (Chen et al., 2018). Consistently, in this study, we found that *MoPCT1* was crucial for vegetative growth, conidiation, appressorium turgor generation and plant infection in the rice blast fungus *M. oryzae*. Interestingly, we also found that *MoPCT1* was involved in cell wall integrity and cell autophagy of the fungus. Therefore, we conclude that CDP-C pathway for PC synthesis plays important roles in fungal development and pathogenicity in *M. oryzae*. To our knowledge, the orthologs of the *MoPCT1* gene has not been functionally characterized previously in phytopathogenic fungi.

Appressorium differentiated from a conidium is a key specialized structure to infect host cells by *M. oryzae* (Howard et al., 1991; Talbot, 2003). In this study, we found that deletion of *MoPCT1* did not affect appressorium formation but significantly reduced appressorium turgor generation (Figures 2A,B). Many researchers have reported that the accumulation of appressorium turgor is related to cell wall integrity, transport and metabolism of nutrients, cell cycle regulation, autophagy, and other processes (Xu et al., 1998; Thines et al., 2000; Veneault-Fourrey et al., 2006; Jeon et al., 2008; Saunders et al., 2010). By staining experiments, we observed that the mobilization and degradation patterns of glycogen stores and lipid droplets during appressorium formation in the *ΔMopct1* were similar to that in the Guy11 strain (data not shown). However, we found that *ΔMopct1* exhibited high sensitivity to cell wall stressors such as Congo red or calcofluor-white (Figure 5), indicating that the *ΔMopct1* displays a significant defect in cell wall integrity. Previously, in *M. oryzae*, it has been shown that the Mps1-MAPK signaling pathway is mainly involved in the regulation of cell wall integrity and appressorium-mediated penetration and infection (Xu et al., 1998; Fujikawa et al., 2009). However, we found that deletion of *MoPCT1* did not affect the phosphorylation level of Mps1 (Supplementary Figure S4). Therefore, how *MoPCT1* affects cell wall integrity in *M. oryzae* remains elusive.

In addition, it has been reported that *MrPCT* negatively regulated autophagy process in *M. robertsii* (Chen et al., 2018). Similarly, we found that the autophagy level of the *ΔMopct1* was significantly higher than that of Guy11 under nutrient-rich conditions (Figure 6A), and the expression of autophagy-related genes, such as *MoATG1*, *MoATG8,* and *MoATG13*, was significantly increased in mycelium of *ΔMopct1* (Figure 6B), indicating that *MoPCT1* negatively regulates autophagy in *M. oryzae*. Previous studies have shown that autophagy is a prerequisite for host infection of pathogen, and the deletion of some non-selective autophagy-related genes can lead to different degrees of reduced pathogenicity or even complete loss of *M. oryzae* (Kershaw and Talbot, 2009).

In the study of two PC metabolic pathways in rats, treatment of rat liver cells with the SAH hydrolase inhibitor 3-deazaadenosine (DZA) inhibited the activity of PE methylated pathway methyltransferases (Cho2 and Opi3), whereas the activity of Pct1 homologous protease increased, suggesting that there may be a compensation mechanism between the two pathways (Pritchard et al., 1982). In this study, exogenous addition of PC did not restore phenotypic defects of the *ΔMopct1* mutant (Figures 7A–D). Meanwhile, ELISA results showed that PC content in the *ΔMopct1* was not decreased compared with wild-type Guy11 (Figure 7E) Therefore, we speculate that blocking of CDP-C pathway may activate PE methylation pathway to maintain proper intracellular PC content level in the *ΔMopct1* mutants. This view was supported by the results of RT-qPCR assay. We found that the expression of the key genes *MoCHO2* and *MoOPI3* in PE methylation pathway was significantly up-regulated in the *ΔMopct1* compared to the wild-type Guy11 (Figure 7F).

In *S. cerevisiae*, the PE methylation pathway is the main methyl consumption pathway, and blocking of PE methylation pathway will lead to accumulation of SAM, resulting in the hypermethylation of histone H3 and phosphatase PP2A (Ye et al., 2017). In this study, we found that the PE methylation pathway was dramatically activated in the *ΔMopct1* (Figure 7F), whereas under CM conditions, the methylation levels of H3 in the *ΔMopct1* was significantly increased at multiple sites, including H3K4, H3K27, and H3K36, compared with Guy11 (Figure 8). Therefore, in *M. oryzae*, even in the case of excessive methyl consumption by the activation of PE methylation pathway, methyl groups were still sufficient for methylation reaction of histone H3. SAM is a major intracellular methyl donor and an intermediate in the methionine metabolic cycle (Stipanuk, 2004; Brosnan and Brosnan, 2006). In this study, LC/MS analysis exhibited that there was no significant difference in the content of methionine cycling-related metabolites, including SAM, SAH and HCY between Guy11 and the *ΔMopct1*, However MET content was significantly increased in the *ΔMopct1* (Figure 9A). Also, we found that the expression levels of methionine cycling-related genes in the *ΔMopct1* were significantly up-regulated under CM conditions, such as *MoMET6*, *MoMET17*, *MoSAM1*, *MoSAH1* (Figure 9B). Among them, *MoMET6*, *MoSAM1*, and *MoSAH1* were also up-regulated during the appressorium infection stage (Supplementary Figure S3B). In contrast, under the MM condition, the expressions of *MoSAM1* and *MoSAH1* in the *ΔMopct1* were not significantly different compared to Guy11, while the expressions of *MoMET6* and *MoMET17* in the *ΔMopct1* were significantly decreased (Figure 9C). These results were consistent with the methylation levels of histone H3 under CM and MM (Figure 8).

The hypermethylation of histone H3 will cause changes in the overall transcription level of genes and affect the metabolism of sulfur-containing amino acids in budding yeast (Ye et al., 2017). Recently, roles of histone H3 methylation in fungal differentiation and pathogenicity have been explored in *M. oryzae* (Pham et al., 2015; Huh et al., 2017; Zhou et al., 2021). Approximately 5% of *M. oryzae* genes showed significant changes depended on H3K4me2 or H3K4me3 during infection-related morphogenesis (Pham et al., 2015). In addition, deletion of *MoJMJ1*, a JmjC-domain-containing histone demethylases encoding gene, led to increased methylation levels of H3K4me3, *ΔMojmj1* showed significant defects in vegetative growth, sporulation, appressorium formation, and infection hypha growth (Huh et al., 2017). In this study, consistently, deletion of *MoPCT1* also resulted in increased methylation levels of multiple sites of histone H3 in *M. oryzae* (Figure 8), which may be one of the causes of phenotypic defects of *M. oryzae*. We speculate that the blocking of CDP-C pathway activates MET metabolism-related cycle and may provide sufficient methyl for H3 hypermethylation. Meanwhile, accelerating MET cycle in the mutant may be an effective way to compensate methyl consumption for the activated PE methylation pathway. However, the exact regulatory mechanisms are still unknown. Taken together, *MoPCT1* plays pleiotropic roles in regulating methionine metabolism and histone H3 hypermethylation to govern *M. oryzae* morphogenesis and pathogenicity.

## 4. Materials and methods

### 4.1. Strains and growth conditions

All the mutants used in this study were generated from *M. oryzae* wild-type strain Guy11. Standard growth and storage procedures for fungal strains were performed as described previously (Talbot et al., 1993). *Escherichia coli* strain T1 (TransGen, Beijing, China) was used for routine bacterial transformations and maintenance of various plasmids in this study.

### 4.2. Gene disruption and complementation

For generating the *MoPCT1* gene replacement vector pKO-*PCT1*, the 1.5 kb upstream and 1.5 kb downstream sequences of target genes were amplified with primer pairs *PCT1*-up-F/R and *PCT1*-down-F/R, respectively. Hygromycin-resistant fragments were amplified by *HPT*-F/R. The PCR products were cloned into pKOV21 vector to generate pKO-*PCT1* (Supplementary Figure S1A). The resulting vectors were transformed into protoplasts of *M. oryzae* Guy11 to generate null mutants, as previously described (Talbot et al., 1993). Hygromycin B (Roche, Mannheim, Germany) was added to a final concentration of 200 μg/ml for transformant selection.

For construction of the complementation vector pHB-*PCT1*, a 1.5 kb native promoter region and 1.9 kb full length region of the *MoPCT1* gene were amplified with primer pairs HB-*PCT1*-F/R. The PCR products were cloned into pCB1532 according to the manufacturer’s instructions of One Step Cloning Kit (Vazyme, Nanjing, China). Then, the plasmid pHB-*PCT1* was transformed into protoplasts of the *ΔMopct1* mutants. Transformants were screened for sulfonylurea resistance on BDCM and verified by PCR.

All primers were synthesized by Sangon Biotech in Shanghai, China, and the sequence of primers is shown in Supplementary Table S1.

### 4.3. Fungal growth, sporulation, and appressorium formation

Vegetative growth was assessed by measurement of colony diameter in plate cultures of different strains grown on different medium at 28°C for 10 days. The level of sporulation was assessed by harvesting conidia from the surface of 10-day-old strains grown on CM solid medium and determining the concentration of the resulting conidial suspension using a hemocytometer (Corning, China).

Appressorium formation of *M. oryzae* requires induction at 25°C in darkness for 24 or 48 h. For the rates of appressorium formation, conidial suspensions (5 × 10^4^ conidia ml^−1^) were placed on hydrophobic coverslips for 24 h and measured by microscopic examination of at least 100 conidia. For inoculation to onion and barley epidermis surfaces *in vitro*, conidial suspensions (5 × 10^4^ conidia ml^−1^) were carefully inoculated on onion or barley epidermis surfaces and incubated for 48 h. Then the appressorium were observed and photographed by optical microscope. Each test was repeated at least three times.

### 4.4. Pathogenicity assay

Two-week-old seedlings of the rice cultivar ‘CO39’ and 7-day-old seedlings of the barley cultivar ‘Golden Promise’ were used for infection assays. For cut-leaf assays, leaf fragments were cut from barley seedlings and placed in plastic plates containing wetted filter papers. Wounded barley leaves were prepared by breaking the cuticle by abrasion with an emery board, and moisturized as same as the cut-leaf assays. Then the conidial suspensions (5 × 10^4^ conidia ml^−1^) were placed onto the leaf surface and incubated at 28°C for 5 days. For spray-inoculation assays, conidial suspensions (5 × 10^4^ conidia ml^−1^ diluted in 0.05% Tween-20) were sprayed on rice or barley plants. Inoculated seedlings were kept in black plastic bags for 24 h and then grown at 25°C and 90% relative humidity for 4 days. Disease lesions were photographed after 5 days of incubation. Each test was repeated at least three times.

### 4.5. Total phosphatidylcholine content

Guy11 and *ΔMopct1* were inoculated on cellophane coated CM solid medium and cultured at 28°C for 10 days. Then same weight of Guy11 and *ΔMopct1* mycelia were collected, and the supernatant was taken as samples after grinding with lysate and centrifuged at high speed. Content of total phosphatidylcholine in samples were analyzed using a PC ELISA kit according to the manufacturer’s protocol (Meilian, Shanghai, China) and measured using a microplate reader (SynergyMx, BioTek, USA).

### 4.6. RT-qPCR analysis

Total RNA was extracted using PureLink RNA Mini Kit (Invitrogen, USA) from mycelia and appressoria. Synthesis of the cDNA and RT-qPCR were performed as described previously (Yue et al., 2017). HiScript® II qRT SuperMix for qPCR (Vazyme, Nanjing, China) were used for reverse transcription. ChamQTM Universal SYBR®qPCR Master Mix (Vazyme, Nanjing, China) were used for RT-qPCR. The relative expression level of each gene was calculated using the 2^-ΔΔCT^ method (LivaK and Schmittgen, 2001). β-tubulin (MGG_00604) was used as an endogenous reference. All the primers used for RT-qPCR assays are listed in Supplementary Table S1. Means and standard deviations were calculated based on three independent experiments.

### 4.7. Western blot analysis

Total protein was extracted from mycelia cultured in liquid CM or MM at 28°C, 180 rpm for 36 h. For measuring the autophagy levels, part of mycelia cultured on CM for 36 h were transferred into liquid MM-N medium cultured for 8 h. Hyphae of each sample were filtered with gauze and washed with sterile distilled water, then squeezed dry for total protein extraction. The extraction of total protein was performed as described previously (Lv et al., 2017). Denatured protein samples were separated on an 10% (15%) SDS-PAGE gel and then transferred to PVDF membrane (Solarbio, Beijing, China). The expression of Mps1 were detected with the anti-p44/42 MAPK antibody (anti-MAPK) and the phosphorylation level of Mps1 were detected with the phospho-p44/42 MAPK antibody (anti-TpEY) (Cell Signaling Technology). The other antibodies used in this work were list in Supplementary Table S2. FDTM FDbio-Femto Ecl chemiluminescent substrate (Fdbio science, Hangzhou, China) was used for antigen antibody detections.

### 4.8. Metabolite extraction and quantitation

The mycelium plugs were inoculated on CM medium covered with cellophane for 10 days, then the surface mycelium was scraped and collected into 2 ml EP tubes and stored at −80°C. The samples were delivered to the company named Clim5 Technology (Hangzhou, China) for the UPLC–MS/MS analysis. There were six replicates per sample.

## Data availability statement

The raw data supporting the conclusions of this article will be made available by the authors, without undue reservation.

## Author contributions

ZX and ZW conceived and designed the experiments, analyzed the data, and wrote the paper. ZX, WL, QT, YX, and ZW performed the experiments. All authors contributed to the article and approved the submitted version.

## Funding

This work was supported by the Natural Science Foundation of China to ZW (Grant No. 32070141).

## Conflict of interest

The authors declare that the research was conducted in the absence of any commercial or financial relationships that could be construed as a potential conflict of interest.

## Publisher’s note

All claims expressed in this article are solely those of the authors and do not necessarily represent those of their affiliated organizations, or those of the publisher, the editors and the reviewers. Any product that may be evaluated in this article, or claim that may be made by its manufacturer, is not guaranteed or endorsed by the publisher.
