# Supplementary material for: Phosphocholine cytidylyltransferase MoPct1 is crucial for vegetative growth, conidiation, and appressorium-mediated plant infection by Magnaporthe oryzae
Source: Front Microbiol. 2023 May 5;14:1136168. doi: 10.3389/fmicb.2023.1136168 (PMC10196169; doi:10.3389/fmicb.2023.1136168)
Supplement: Supplementary file 1 [file Data_Sheet_1.docx]

Supplementary Material

Phosphocholine cytidylyltransferase MoPct1 is crucial for vegetative growth, conidiation and appressorium-mediated plant infection by *Magnaporthe oryzae*

Zhe Xu, Qi Tong, Wuyun Lv, Yu Xiao and Zhengyi Wang*

*** Correspondence:** Zhengyi Wang: zhywang@zju.edu.cn

# Supplementary Figures and Tables

## Supplementary Figures


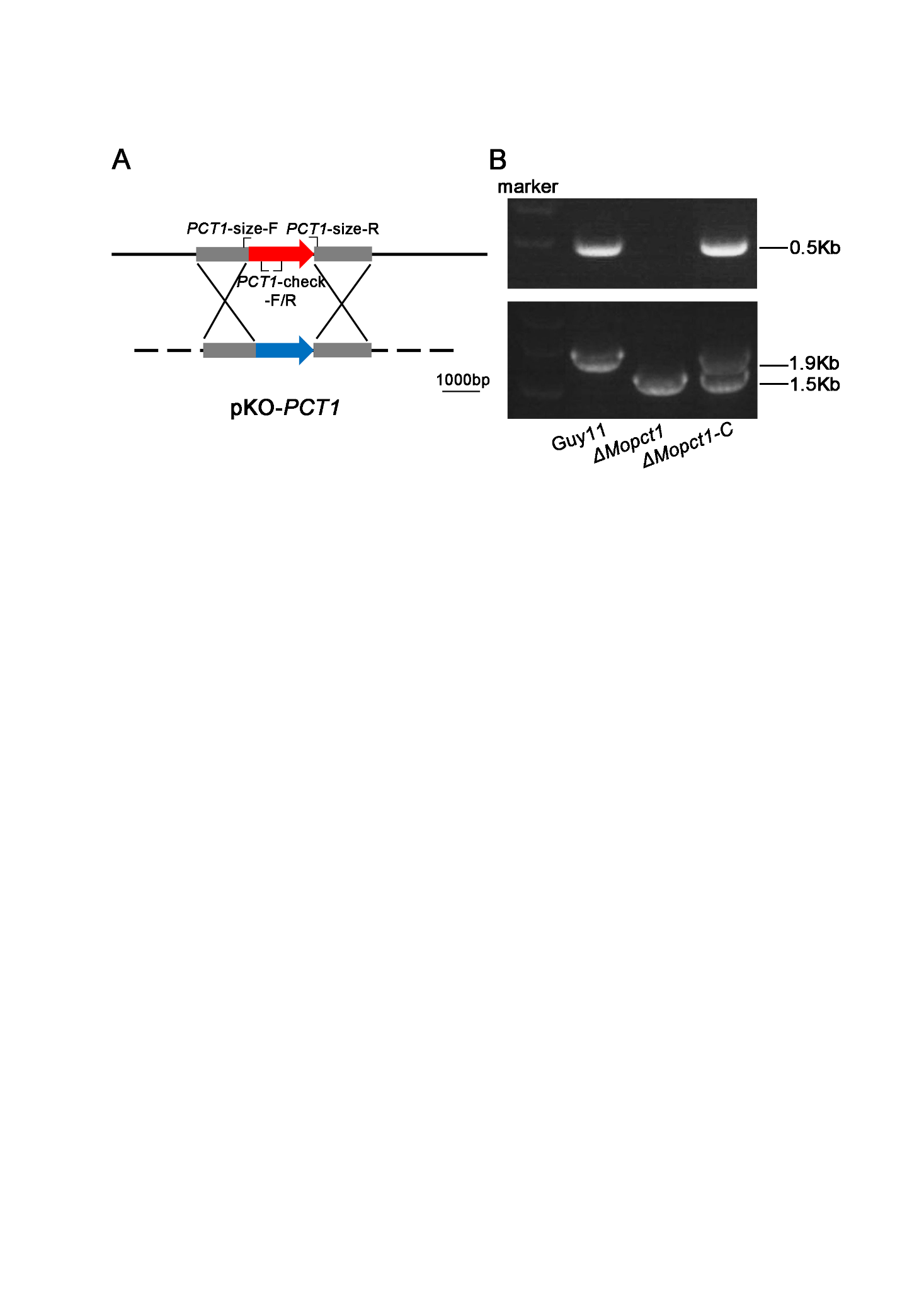


**Figure S1.** **Targeted gene replacement of *MoPCT1* and confirmation of the null mutant and the complemented strain by PCR.** **(A)** Construction of the knockout vector pKO-*PCT1* and the location of primers used for *ΔMopct1*. Red arrow represents the target gene *MoPCT1*. Blue arrow represents the hygromycin resistant gene. **(B)** Confirmation of the null mutant and the complemented strain by PCR. Upper: W/WO the targeted gene amplified by internal primers *PCT1*-check-F/R; Lower: size difference of DNA amplified by primers *PCT1*-size-F/R.


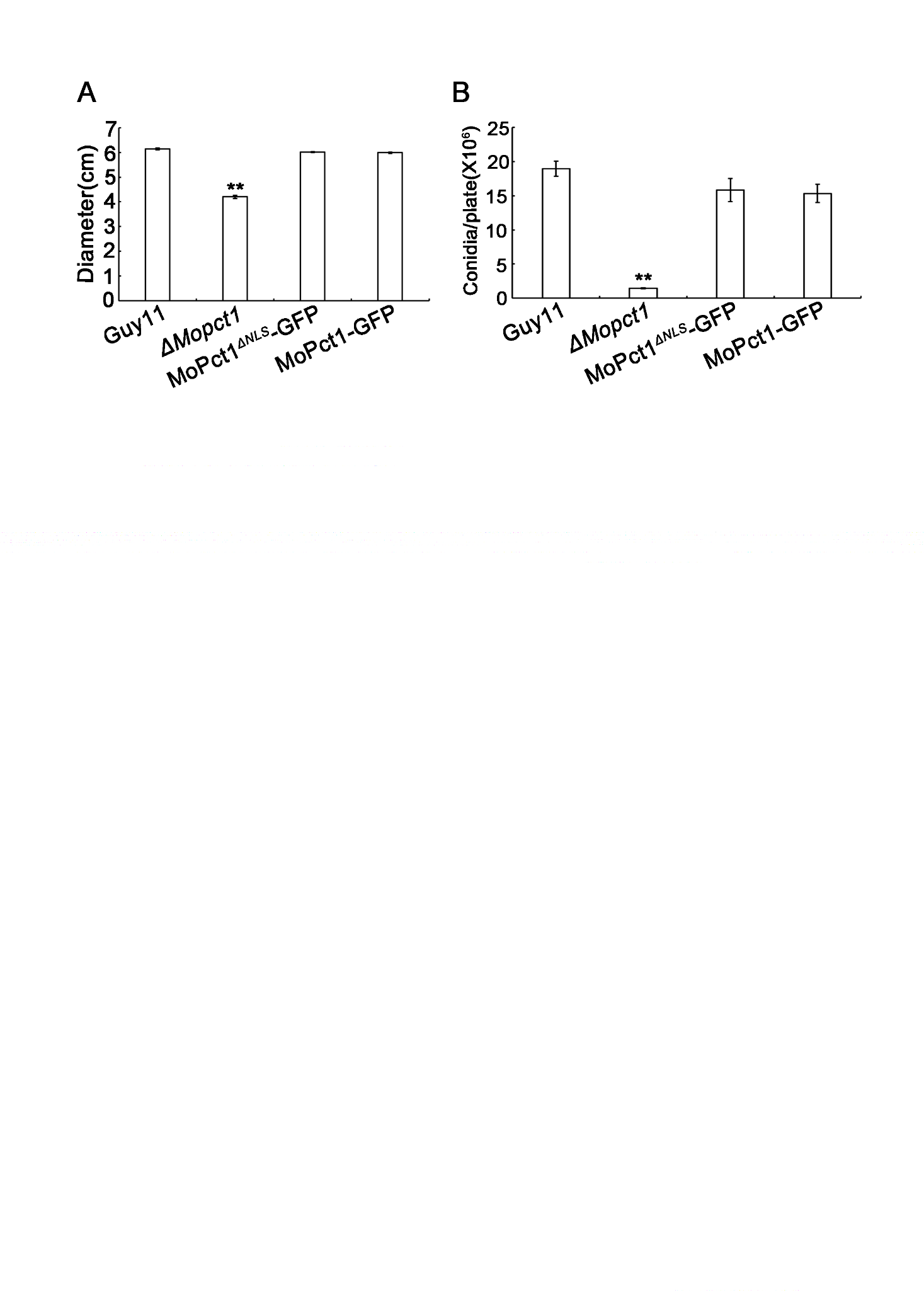


**Figure S2. NLS sequence is not important for vegetative growth and sporulation of *M. oryzae*. (A)** Radial growth of each strain on CM plates. **(B)** Statistical analysis of conidiation. Error bars represent standard deviation. Double asterisks indicate significant difference (P＜0.01).


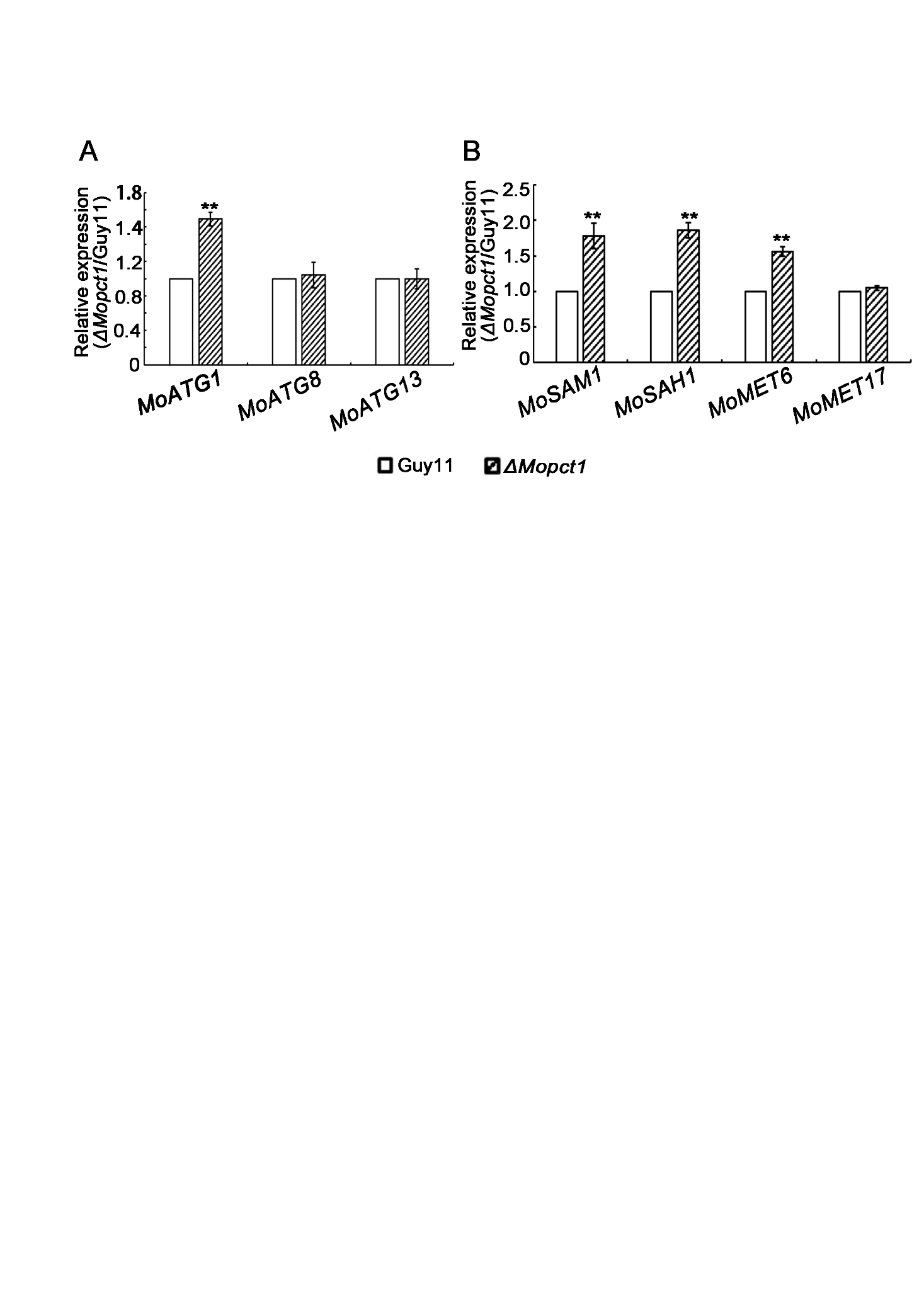


**Figure S3. Expression levels of pathogenicity-related genes during appressoria infection stage.** **(A)** Expression levels of autophagy-related genes. **(B)** Expression levels of the genes associated with methionine metabolic cycle. Total RNA was extracted from appressorium induced on barley cut-leaf for 24 hours and determined by RT-qPCR. Error bars represent standard deviation. Double asterisks indicate significant difference (P＜0.01).


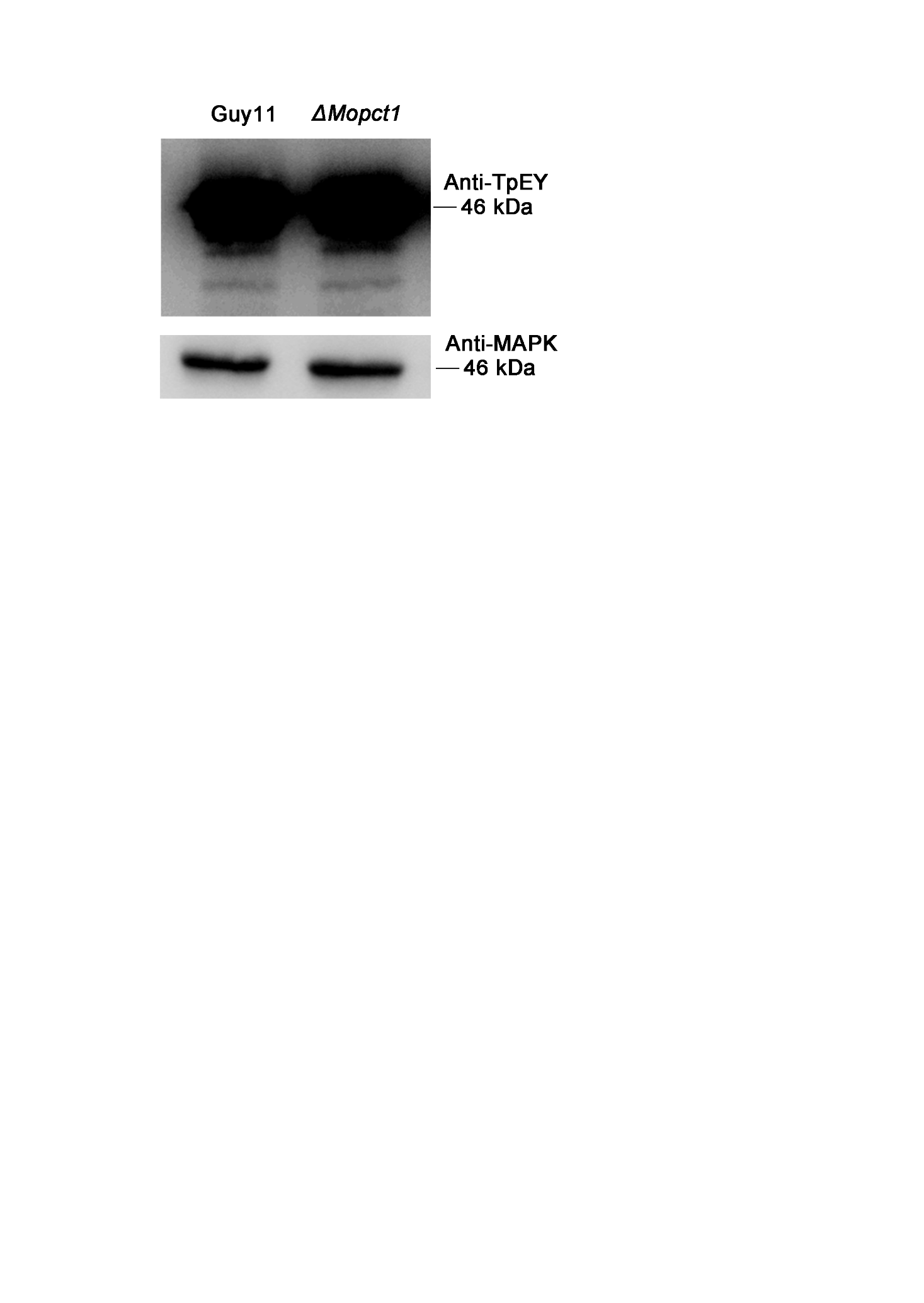


**Figure S4. MoMps1 phosphorylation assays.** Total proteins were isolated from mycelia of each strain. The anti-MAPK and anti-TpEY antibodies were used for the expression and phosphorylation level of MoMps1 (46 kDa), respectively.

## Supplementary tables

**Table S1. Primers used in this study**

| **Name** | **Sequence (5’-3’)** | **Application** |
| --- | --- | --- |
| HPT-F | GACAGACGTCGCGGTGAGTT | Gene disruption and complementation |
| HPT-R | GTCCGAGGGCAAAGAAATAG |  |
| *PCT1*-up-F | CCCCCGGGCTGCAGGAATTCCGCCTAACACAAACACCTG |  |
| *PCT1*-up-R | TCCTTCAATATCATCTTCTCTCGTGTCTGGTCCGATGTGCGTC |  |
| *PCT1*-down-F | TAGATGCCGACCGAACAAGATGAGAGGGTGCAATGTTGGA |  |
| *PCT1*-down-R | TACCGGGCCCCCCCTCGAGTTCCGACACCTCCGCACATC |  |
| *PCT1*-check-F | GCCATCCAGAGGTCTCAAAT |  |
| *PCT1*-check-R | ATGTCCATCCCAATGTGC |  |
| *PCT1*-size-F | CCTTTGCTACCATACGTACACA |  |
| *PCT1*-size-R | CTTCTTTGCCTCCGTCCCAGTA |  |
| HB-*PCT1*-F | TCCCCCGGGCTGCAGGAATTCCGCTTAGTCTCTAGTAGC |  |
| HB-*PCT1*-R | GATAAGCTTGATATCGAATTCCTGCGGATCCCCTAACGAT |  |
| *PCT1*-GFP-F | GGATCCCCCGGGCTGCAGGAATTCCCAGTCTCGCTTAGTCTCTAG | Construction of NLS related vectors |
| *PCT1*-GFP-R | GCTCCTCGCCCTTGCTCACCATGGCTCCTGTAGCAGC |  |
| NLS-GFP-F1 | Same as PCT1-GFP-F |  |
| NLS-GFP-R1 | CGACGGCATTGTTGCTCGACGGGTGCGAGGTGGGTG |  |
| NLS-GFP-F2 | TCGAGCAACAATGCCGTCG |  |
| NLS-GFP-R2 | Same as PCT1-GFP-R |  |
| βtub-qRT-F | CATACGGTGACCTGAACTAC | RT-qPCR for mycelia |
| βtub-qRT-R | CCATGAAGAAGTGCAGACG |  |
| *CHO2*-qRT-F | ATGAGGACTTGGACGAGGAG |  |
| *CHO2*-qRT-R | GGACTTGTTTGGCATTGC |  |
| *OPI3*-qRT-F | CTCCTCGACAATGACCTAGCG |  |
| *OPI3*-qRT-R | CAACACCCAAGCCGTCAACC |  |
| *PCT1*-qRT-F | CATCTACGGACCCATCAAG |  |
| *PCT1*-qRT-R | CAACTCGTTCTTCTTCAGCC |  |
| *PSD1*-qRT-F | ATCCAACAAGACCGACCA |  |
| *PSD1*-qRT-R | AGATGACGGCGTAGTAGAGC |  |
| *PSD2*-qRT-F | GCAAACCAAAGACCATCG |  |
| *PSD2*-qRT-R | CATACCACCATAACCCGAC |  |
| *MET6*-qRT-F | TGAAGGGTATGTTGACGGG |  |
| *MET6*-qRT-R | TCAGAGTAGCAGAAGTGCGA |  |
| *MET17*-qRT-F | CGGCTTCAAGATGATTAGC |  |
| *MET17*-qRT-R | ACTGCTCAAAGTCGGCAA |  |
| *SAH1*-qRT-F | CAAGAGCGTCCAGAACATC |  |
| *SAH1*-qRT-R | GGTTGGTAAAGGAGCAGGA |  |
| *SAM1*-qRT-F | CCAGATTATCCGTGACAACTTC |  |
| *SAM1*-qRT-R | AGTGACCGTTCTTGGCAGT |  |
| *ATG1*-qRT-F | GGCGAGGAAGATGTTCTCA |  |
| *ATG1*-qRT-R | GATAGTGTCTGCGTGTTCACA |  |
| *ATG8*-qRT-F | CATTCCCGTCATTTGCGA |  |
| *ATG8*-qRT-R | TCCGTCCTCGTCCTTGTGTA |  |
| *ATG13*-qRT-F | AACCGCAGCATCAGTCTT |  |
| *ATG13*-qRT-R | CCCAAACTACCAGTGAACCT |  |
| *β-tub*-AP-F | CTGCTTTCTGGCAAACTATC | RT-qPCR for appressoria |
| *β-tub*-AP-R | TGAAGTAGACGCTCATACGC |  |
| *ATG1*-AP-F | GGCGAGGAAGATGTTCTCA |  |
| *ATG1*-AP-R | GATAGTGTCTGCGTGTTCACA |  |
| *ATG8*-AP-F | CATTCCCGTCATTTGCGA |  |
| *ATG8*-AP-F | TCCGTCCTCGTCCTTGTGTA |  |
| *ATG13*-AP-F | GGTCGCAGAGACTCCTTTAC |  |
| *ATG13*-AP-R | GAATCAGTATCCGAGCCAG |  |
| *SAM1*-AP-F | CCAGATTATCCGTGACAACTTC |  |
| *SAM1*-AP-R | AGTGACCGTTCTTGGCAGT |  |
| *SAH1*-AP-F | GGCCAACGGCCGTCACATCA |  |
| *SAH1*-AP-R | TCGGCCTGGACGGGGGTAAG |  |
| *MET6*-AP-F | TGAAGGGTATGTTGACGGG |  |
| *MET6*-AP-R | TCAGAGTAGCAGAAGTGCGA |  |
| *MET17*-AP-F | AATACCCACCTTGCCACAC |  |
| *MET17*-AP-R | GGTTTCAAAGTTCTTGCTCAGG |  |

**Table S2. Antibodies used in this study.**

| **Antibody** | **Supplier** |
| --- | --- |
| Anti-GFP antibody (M20004) | Abmart |
| Anti-GAPDH antibody (EM1101) | HUABIO |
| Goat anti-Rabbit IgG-HRP (HA1001) | HUABIO |
| p44/42 MAPK (Erk1/2) (137F5) Rabbit mAb | Cell Signaling Technology |
| Phospho-p44/42 MAPK (Erk1/2) (Thr202/Tyr204) (D13.14.4E) XP® Rabbit mAb | Cell Signaling Technology |
| Anti-H3 antibody (PTM-1001) | PTM BIolabs |
| Anti-H3K36me antibody (PTM-623) | PTM BIolabs |
| Anti-H3K36me2 antibody (PTM-624) | PTM BIolabs |
| Anti-H3K36me3 antibody (PTM-625) | PTM BIolabs |
| Anti-H3K4me antibody (PTM-611) | PTM BIolabs |
| Anti-H3K4me2 antibody (PTM-612) | PTM BIolabs |
| Anti-H3K4me3 antibody (PTM-613) | PTM BIolabs |
| Anti-H3K27me2 antibody (PTM-5010) | PTM BIolabs |
| Anti-mouse IgG,HRP-linked antibody (7076S) | Cell Signaling Technology |
